# Optical coherence tomography monitoring and diagnosing retinal changes in multiple sclerosis

**DOI:** 10.1002/brb3.2302

**Published:** 2021-09-14

**Authors:** Arshad Mehmood, Wajid Ali, Shuang Song, Zaheer Ud Din, Ruo‐Yi Guo, Wahid Shah, Ikram Ilahi, Bowen Yin, Hongjing Yan, Lu Zhang, Murad Khan, Wajid Ali, Liaqat Zeb, Hamidreza Safari, Bin Li

**Affiliations:** ^1^ Department of Neurology The Second Hospital of Hebei Medical University City Shijiazhuang Hebei Province P. R. China; ^2^ Key Laboratory of Neurology of Hebei Province City Shijiazhuang Hebei Province P. R. China; ^3^ Key Laboratory of Functional Inorganic Materials Chemistry School of Chemistry and Materials Science, Heilongjiang University Harbin P. R. China; ^4^ Institute of Cancer Stem Cell Dalian Medical University Liaoning Province P. R. China; ^5^ Department of Physiology Hebei Medical University Shijiazhuang Hebei P. R. China; ^6^ Department of Zoology University of Malakand Chakdara Khyber Pakhtunkhwa Pakistan; ^7^ Department of Neurology The First Hospital of Qinhuangdao Qinhuangdao Hebei P. R. China; ^8^ Department of Genetics Hebei Key Lab of Laboratory Animal Hebei Medical University Shijiazhuang Hebei Province P. R. China; ^9^ Green and Environmental Chemistry, Ecotoxicology and Ecology Laboratory, Department of Zoology University of Malakand Chakdara Khyber Pakhtunkhwa Pakistan; ^10^ School of Bioengineering Dalian University of Technology Dalian Liaoning P.R. China; ^11^ Department of Immunology Torbat Jam Faculty of Medical Sciences Torbat Jam Iran

**Keywords:** experimental autoimmune encephalomyelitis, ganglion cell layer, multiple sclerosis, optical coherence tomography, retinal nerve fiber layer

## Abstract

This study explores the use of optical coherence tomography (OCT) to monitor and diagnose multiple sclerosis (MS). The analysis of reduced total macular volume and peripapillary retinal nerve fiber layer thinning are shown. The severity of these defects increases as MS progresses, reflecting the progressive degeneration of nerve fibers and retinal ganglion cells. The OCT parameters are noninvasive, sensitive indicators that can be used to assess the progression of neurodegeneration and inflammation in MS.

## 1 INTRODUCTION

Multiple sclerosis (MS) is a neurodegenerative and inflammatory disease of the central nervous system (CNS). The disorder involves axonal loss and demyelinating lesions related to the demise of the oligodendrocytes, astrogliotic proliferation, and neurodegeneration. Multifocal CNS damage is often spread over time, resulting in significant neurological abnormalities and disabilities. However, neurodegeneration and inflammation play a key role in MS, while both processes differ in the dynamics. The inflammatory processes in the early stages of the disease are dominant. They slow down as the disease progression accelerates axonal degeneration, which is the primary cause of MS‐associated defects (Frohman et al., 2008; Polman et al., 2011; Trapp et al., 1998). MS neurological signs are not pathognomonic for final diagnosis. The key criteria for diagnosis include the reported multifocal lesions and the onset of the individual symptoms at various times and spaces. The presence of cerebrospinal fluid oligoclonal bands, irregular stimulated potential lesions, and multifocal lesions in magnetic resonance imaging (MRI) provides a faster diagnosis. The interdependency among diffuse inflammation, focal inflammation, neurodegeneration, and their relative involvement in clinical deficiencies remains uncertain (Huang et al., 1991). Since oligodendrocyte defect would contribute to MS pathology, it should be eliminated to prevent axonal degeneration.

OCT is a noninvasive technique employed in MS as a possible marker for axonal retinal degeneration. The most commonly evaluated OCT parameters are the total macular volume (TMV) and retinal nerve fiber layer (RNFL). This study analyzes retinal changes in MS patients and in the most frequently used animal model of demyelination including experimental autoimmune encephalomyelitis (EAE). Subsequently, this review briefly explains how OCT and other imaging tools have improved our ability to monitor and diagnose retinal changes in MS.

## 2 RETINA AND THE OPTIC NERVE

The retina is a unique tissue in the sensory system of living organisms. Developmentally, it is linked to the cerebrum, as it differentiates from the two layers of the optic cup, which is the prominence of the neural tube. Therefore, considering the retina is a part of the CNS, it can be classified into two fundamental parts: first is neuroepidermal, which include cones and rods, while the second is a cerebral one, which includes the ganglion and bipolar cells comprising the second and third neurons of the optic tract. The nerve fiber layer is formed by the axial extensions of ganglion cells, stretch toward the optic disc, where they leave the eye as an optic nerve comprising 1.0‐1.2 million nonmyelinated axons of retinal ganglion cells (RGCs). It enters the orbit via the scleral lamina cribrosa, where the myelin sheaths of the nerve fibers originate. Histologically, the optical nerve, which comprises axons of ganglion cells, microglia, oligodendrocytes, and astrocytes, indicates a higher similarity to the white matter of the brain instead of the peripheral nerve (M. Wojtkowski et al., 2002). RNFL contains only the axons of glia cells and ganglion, even without myelin sheath, which leads to its uniqueness. Furthermore, the measurement of RNFL thickness monitors the actual axonal injury and does not affect the results through the thickness and presence of the myelin sheath. However, the RNFL can be the location of preference for screening and evaluation of neurodegeneration.

Neuronal and axonal impairments are commonly considered crucial incidents in the progression of MS diseases. In clinical practice, patients presenting with the first clinical incident strongly predictive of MS are usually diagnosed with the clinically isolated syndrome (CIS). Oberwahrenbrock et al. demonstrated that retinal neurodegeneration is observable but independent and dependent on clinical relapses such as optic neuritis (ON) in CIS patients (Oberwahrenbrock et al., 2012). This study is the first to evaluate changes in the intraretinal layer or to diagnose retinal neurodegeneration independent of ON in a larger population of CIS patients. It is suggested that the thalamus can be the site of early neurodegeneration in MS. Moreover, findings established the existence of thalamic atrophy in radiologically isolated syndrome, which is usually a sign of early CNS demyelinating disease, and it must be studied as a neurodegeneration‐associated metric (Azevedo et al., 2015).

Postmortem findings reveal pathology of the retina in MS beyond injury to the ganglion cell layer (GCL) and RNFL. Furthermore, retinal pathology could not only progress as a cause of inflammatory attack to the anterior optic pathway, inducing retrograde neuronal and axonal impairment with thinning of retinal GCL and RNFL, but the retina can be a primary target of inflammatory or degenerative processes. However, OCT is frequently used as a marker for axonal loss in treatment trials of MS (Green et al., 2010).

## 3 MULTIPLE SCLEROSIS

In MS patients, disturbance of the inner retina (slits), optic disc pallor, and retinal periphlebitis can be shown through funduscopic examination (Frisén & Hoyt, 1974; Rucker, 1972). The pale optic disc indicates the process of atrophy in the peripapillary RNFL (pRNFL) (Frisén & Hoyt, 1974). RNFL disturbances are functional in visual function declines. As previously reported, the molecular and cellular mechanisms underlying retinal alterations in the demyelinating disease may be effectively understood by immunohistochemical and histological examination. Unfortunately, there is restricted availability of applicable tissue for assessment in the evaluation of human disease demyelinating injury. In MS, the bulk of tissue samples come from brain biopsies and autopsy specimens at the end of the disease episodes or end of life for acute fulminant episodes that are caused by extreme inflammation. However, these samples do not have unimpeachable results on the time period of the demyelinating mechanism. Moreover, in MS, the retinal pathology study mostly investigates a limited number of instances and does not present a deep clinical background of the reported patients. In 1983, Toussaint et, al. reported 32 cases of MS in a pathological test includes 15 cases in which the retina was examined (Toussaint et al., 1983). Among the 15 patients, the material of abnormal fibrillar and retinal veins material was found in six patients, without the connection with the period of the disease. This frequently thickened content, stained green with Masson's trichrome, and unstained with glial fibrillary acidic protein, was spread across the retinal veins, from the peripapillary region to the equator. Rarefaction of macular RGCs and a decrease in the number of RNFL axons were identified as visible in most evaluated eyes. Intriguingly, in two of these specimens, several nodular lymphoplasmacytoid infiltrates encircling the retinal vessels were reported including one which invades the vitreous (Kerrison et al., 1994). Pathological retinal assessments of 29 patients (26 MS, 3 neuromyelitis optica [NMO]) were presented mainly focused on the retinal vasculature. The initial assessment of the vessel in the histopathological portion in the horizontal plane through the pupil and the optic nerve indicated one case of inflammation (among 26) while investigating the trypsinized vascular network of caps, inflammation was obvious in 20% of instances (4 out of 20), while 3 of them were bilateral. Atrophy of the GCL and RNFL were present in 73% of cases. There was no evidence of venous sclerosis, while choroiditis was found in 11.5% of cases. In 2001, it was reported that axonal loss pattern and cell‐specific reduction in the neuronal density presented guidance into the pathological process of MS by investigating the autopsy‐collected tissue of a lateral geniculate nucleus, optic tract, and optic nerve (Evangelou et al., 2001). It is reported that patients with MS showed smaller mean cross‐sectional areas in the optic tract as well as the optic nerve and substantially decreased axonal density via the preparations of imaging tissue through a pixel intensity cluster program to measure axons. In the lateral geniculate nucleus of the affected patients, neuronal density was substantially decreased, and the susceptibility size of axons was shown in the anterior visual pathway to injury. In MS patients with or without ON, pathological modification of tissues was identified in the retina (macular and peripapillary), iris, optic nerve head, and optic disc (Blumenthal et al., 2009; Costello et al., 2006; Trip et al., 2005). Moreover, it is conducted that a systematic retinal pathology assessment of 82 patients in MS, which confirmed that retinal inflammation is prominent and prevalent in disorder (particularly perivascularly). The concerned vessels are located within the nerve fiber layer of the retina (in MS, the primary layer of interest). The mononuclear lymphocytes can be found near the retinal vasculature, while HLA‐DR positive cells may be found in the inner nuclear layer (INL), RGC layer, iris, optic nerve, and optic disc. The proliferation of astrocytes could be found in the optic nerve head and around the vessels. In addition, both inflammation and tissue atrophy could be observed in the deeper layers of the retina (including INL). Furthermore, optic disc astrocytic gliosis, optic nerve, and findings of gliomesodermal reaction may also be predicted to be found. Subsequently, confocal Z01 microscopy, occludin protein‐stained, optic, and retinal nerve vasculature preparations indicate a tight junction opening that can be linked with the glial cell reactions. In the eyes of MS patients, morphological changes also showed that the optic discs appear cupped or indented (Green et al., 2010). However, this analysis shows several drawbacks such as the limited group of eyes (eight) evaluated with immunohistochemistry and the lack of past clinical history, the analysis of INL atrophy along with the reduced involvement of mononuclear cells generated valid evidence in support of retrograde trans‐synaptic degradation, validating previous findings indicating this phenomenon (Audoin et al., 2006; Reich et al., 2009).

The fact that the human retina is commonly free of myelin complicates the comparison between retinal pathology and the brain. Despite this drawback, the study of neuronal disruption and various patterns of inflammation among various anatomical structures have great significance in achieving a comprehensive knowledge of the pathophysiology of the disease. Active white matter demyelinating lesions are represented by the existence of macrophages which contain cytoplasmic early myelin degeneration materials both during the lesion and at the lesion edge (Brück et al., 1995). All these lesions are highly uncommon in chronic MS and frequently present in the active form of MS (secondary progressive MS [SPMS] and relapsing‐remitting MS (RRMS) with relapses), which is a potentially pathological substrate for the attacks (Lucchinetti et al., 2000). In brain lesions, perivascular inflammation is commonly described as containing granulocytes, plasma cells, fewer B lymphocytes, as well as T lymphocytes, are typical characters to MS retina, where the vessels running via the RNFL are known as indicating a perivascular inflammation involving mononuclear cells, lymphocytes, and phagocytic (Popescu & Lucchinetti, 2012). Localized perivascular infiltrate within the retina is more common in SPMS as well as RRMS (29%) than in primary progressive MS (PPMS) (5%) and acute demyelinating lesions (Green et al., 2010). It is suggested that these results reflect the hypothesis that the immune response might also be targeted toward certain other antigens, not linked to myelin (Petzold et al., 2010).

In the MS brain, neuronal disruption has been identified as a result of acute local inflammation, which decreases with the lesion chronicity (Frischer et al., 2009; Kuhlmann et al. 2002).  However, in the active MS plaques, the axonal loss is most extreme, followed by normal‐appearing white matter, inactive plaques, and smoldering lesions (Lassmann, 2003; Trapp et al., 1998). To a great extent, cell depletion is present at a lower stage in PPMS and SPMS than in RRMS (Frischer et al., 2009). In comparison, a decrease in the RGCs population was recorded in 79% of chronic MS retinal specimens, while depletion of RGC was apparent in 55% of the eyes when the instances were categorized as acute (Petzold et al., 2010). This evidence indicates that already reported pronounced diffuse inflammatory response during the development of the disease in normal‐appearing white matter (perivascular cuffs of mononuclear cells, diffuse infiltration of tissue by profound microglial activation, and T lymphocytes) can have similar effects of chronic retinal inflammation reported in progressive MS cases (Kutzelnigg et al., 2005).

In healthy populations, OCT imaging of RNFL is approximately equal to histological evaluation (Blumenthal et al., 2009; Green et al., 2010). As previously reported, it is well known that MS people develop RNFL loss, regardless of the diagnosis of ON. Using OCT, different groups reported regarding the thinning of RNFL in the eyes of MS patients without previous ON (Costello et al., 2009; Fisher et al., 2006; Parisi et al., 2001; van Dijkman et al., 2018). The introduction of OCT to the research of cutting‐edge MS now provides more appropriate illustrations of the structure‐function relationship in identifying the pathophysiology of this puzzling disorder (Petzold et al., 2010). In diseases, such as MS, the loss of myelin leads to nerve signal transmission disturbance, axonal damage, and neurodegeneration. In MS, this chronic mechanism emerges to describe and anticipate short‐ to medium‐term impairment (Cordano et al., 2019; Martinez‐Lapiscina et al., 2016). Some subtypes of axons/neurons reveal the enhanced relative susceptibility to loss in chronic disease, particularly those with soma size and small axonal diameter (DeLuca et al. 2004). In a meta‐analysis, RNFL loss due to neurodegeneration was measured as 7 μm (average), while RNFL loss per year was reported to be 2 μm in 299 patients for approximately 4 years (Petzold et al., 2010). Intriguing studies consisting of OCT and MRI have explored the mechanisms behind the reported chronic axonal loss. It is analyzed that relationship between disruption in the posterior and anterior visual pathways, the presence of anterograde and retrograde trans‐synaptic destructions, throw volumetric calculation of the synaptic relay between the posterior as well as anterior pathway (thalamus), OCT, and optic radiation lesions (Gabilondo et al., 2014). In the cohort of primary progressive MS patients, the association of cortical lesions, as well as OCT in MRI (phase‐sensitive inversion recovery and double inversion recovery sequences), indicated a relationship of the cortical lesions with macular volume (MV) and macular ganglion cell‐inner plexiform layer (GCIPL), while not with RNFL (Petracca et al., 2017). Recently, it is reported that models based on MRI metrics and OCT can predict visual impairment evaluated with low‐contrast letter acuity.

Vacuolar macular changes, primarily in the INL of the perimacular rim (Saidha et al., 2012), are observed in 0.8−6% of patients with MS (Balk et al., 2012; Gelfand et al., 2012; Saidha et al., 2012), in particular those with acute ON history, and in 20% of patients with NMO, where microcystic macular edema (MME) is identified mostly in prior ON eyes (Green et al., 2013). Furthermore, MME is not specific to the demyelinating disease and has already been identified in trauma, hydrocephalus, prominent vitreous traction, compressive optic neuropathy, tamoxifen retinopathy, age‐related macular edema (ME), glaucoma, dominant optic atrophy, neurofibromatosis 1‐associated optic nerve glioma, Leber's hereditary optic atrophy, and group 2A idiopathic juxtafoveolar retinal telangiectasis (Abegg et al., 2014; Barboni et al., 2013; Gaudricet al., 2006; Wolff et al., 2013). However, it has been linked to INL microglial activation results in the breakdown of the blood‐retinal barrier (Gelfand et al., 2012), while some others have hypothesized that MME might be the cause of Müller cell disruption (Balk et al., 2012) or trans‐synaptic retrograde deterioration (Abegg et al., 2014; Vanburen, 1963). These kinds of hypotheses might be compatible with various contributing processes acting on patient‐specific factors (Green et al., 2013). In patients with MS, a worsening impairment and disease progression were linked to the presence of MME, lower visual acuity (Gelfand et al., 2012; Saidha et al., 2012), a thin retinal nerve fiber layer (Gelfand et al., 2012), and increased thickness of INL (Saidha et al., 2012). The INL volume represents immunotherapy‐response (Knier et al., 2016) and could be an effective process for patients with progressive MS stratification to choose treatment (Cellerino et al., 2019). INL swelling tends to be the most common during early disease, although thickening decreases later until the swelling starts with atrophy (Cordano et al., 2021). In addition to MME, the ME can be detected in the eyes of MS patients when followed by comorbid clinical uveitis, or as a result of fingolimod, a sphingosine‐1‐phosphate receptor (S1PR) modulator, which was the first FDA‐approved oral drug to treat the relapsing types of MS. Uveal tract inflammation can be detected in 1–3% of MS patients and 10 times as prevalent in patients with MS as compared to the general population. It is normally intermediate (pars planitis), bilateral as well as chronic, while it may be complicated with the retinal neovascularization or detachment, cystoid ME, and epiretinal membrane formation (Le Scanff et al., 2008). Intriguingly, about 10% of intermediate uveitis patients develop MS. Some MS therapies, including fingolimod, may also lead to cystoid ME. ME is detected in 0.5% of patients with MS treated with fingolimod, also with a dose‐dependent incidence, and usually ceases after the discontinuation of treatment (Jain & Bhatti, 2012). Since the S1PR acts on intercellular junctions and cell cytoskeleton (Dudek et al., 2004), it controls the integrity of the endothelial barrier. ME associated with fingolimod can be induced by the breakdown of the internal retinal blood barriers via receptor internalization (Sanna et al., 2006).

In this context, it is worth mentioning that recent works indicate the visuomotor system might be applied to control the motor disability in MS by monitoring fixational microsaccades (Serra & Petzold, 2020; Sheehy et al., 2020).

## 4 OPTIC NEURITIS IN MULTIPLE SCLEROSIS

ON is an inflammatory episode in the optic nerve, resulting in conduction block, demyelination, and consequent subacute vision loss. It is usually found in RRMS in approximately 70% of patients, while the symptom is present in about 25% of patients (Toosy et al., 2014). ON is the prominent aspect of the less frequent NMO as well as myelin oligodendrocyte glycoprotein (MOG) antibody‐associated disease, while it can develop in combination with Lyme disease, vasculitis, acute disseminated encephalomyelitis, syphilis, and sarcoidosis. ON can also be induced via immunization or viral illness. The axonal defect is a less frequent but well‐known hallmark of demyelinating lesions, with a strong association of both transected axons and the degree of inflammation in the lesion (Trapp et al., 1998). The causes and timing of axonal loss underlying demyelinating injuries are unknown. Direct defect from glial/immune cells (Ferguson et al., 1997; Kornek et al., 2000; Trapp et al., 1998), secondary ischemic injury, and loss of trophic support from myelin have been involved, and each can be operating to a different degree. In acute transection, studies of animals have shown that die back to the RGC soma can take few months (Quigley et al., 1977). The symptoms and retinal degree of retrobulbar demyelinating injury are mainly depending on the position of the main lesion. Evidently, in ophthalmological evaluation, the acute phase indicates that up to two‐thirds of patients have a normal optic disc (retrobulbar ON), while a third patient shows a swollen optic disk (papillitis, bulbar ON) (Fisher et al., 2006). Delay of the p100 visual evoked potentials, asymmetry, alteration of the wave morphology along with related pain, movement of the eye, relative pupil afferent pupillary damage, optical nerves MRI, deficits on kinetic visual field and static test, which following the RNFL topography, with the consequent central, altitudinal, cecocentral, and arcuate deficits. Evaluation on low‐contrast visual sensitivity and color discrimination are approaches that can assess whether a patient has an acute inflammatory ocular event undergo a regular funduscopic investigation (Galetta & Balcer, 2018; Keltner et al., 1993; Tintoré & Montalban, 2018).

The optic nerve damage is more frequent than clinically observable incidents, with about 100% of patients experiencing anterior visual pathway demyelination at the end of life (Toussaint et al., 1983).

OCT, a noninvasive imaging technique that has already been applied to develop quantitative methods for assessing the inner retina, has created crucial advancements in our knowledge about the neurodegeneration and inflammation timing of MS ON (Bischof et al., 2017). It was discovered almost 20 years ago that pRNFL and MV were abnormal in ON, using the time‐domain (TdOCT) (Parisi, 2003; Trip et al., 2005). The pRNFL, which reflects the intraocular, nondemyelinated axons of the RGCs all‐around optic nerve head, is substantially decreased 3 months after the acute event, and also the thinning terminates about 6 months after the acute event (Henderson et al., 2010; Klistorner et al., 2010). MS and ON have a significant effect on the temporal quadrant of the retina, which contains the papillomacular bundle, that is responsible for central vision (Saidha et al., 2012). The papillomacular bundle is primarily composed of parvocellular axons. A potential postulate indicates that the size‐selective loss is a result of the failure of small axons to remyelinate. After a single ON episode, the thinning of RNFL is about 5–40 μm (average 10–20 μm) (Costello et al., 2006, 2008). In comparison, eyes with more than one previous ON indicate a considerable thinning when compared to the single ON (Costello et al., 2006). OCT shows more RNFL swelling during acute retrobulbar ON than can be observed in routine fundoscopy with a swelling up to 82% (Kupersmith et al., 2011), obviously due to axoplasmic flow stasis. Currently, MV has been implemented to quantify the integrity of RGCs (macular GCIPL) by incorporating segmentation algorithms (Petzold et al., 2017). In acute phases, GCIPL assessment is a more effective method for tracking axonal loss, possibly the effect of remyelinating/neuroprotective drugs when compared to pRNFL, being less affected via axonal swelling due to edema and inflammation. At the clinical onset, pRNFL edema is present and almost absent in the macula while it disappears after 1 month. Volumetric examination of the retinal layers and macula shows a biphasic pattern, specified via atrophy of inner retinal layers (macular RNFL [mRNFL] and combined GCIPL). However, in the first 2 months, swelling of the outer layers followed by reduction of edema in the outer layers and gradual neurodegeneration of GCIPL and mRNFL in the next 4 months. As predicted, GCIPL thinning is the best predictor for short‐term visual deficit effects such as vision with low contrasts, color vision recovery, and visual fields (Gabilondo et al., 2015).

However, there is a lack of certainty about the real meaning (axonal loss and edema/cell infiltration rather than neuronal atrophy) of the stated acute thickening and subsequent subacute thinning of the retinal layers. Regarding these aspects, the monitoring of retinal changes is due to the ON provides a valuable way to evaluate the treatment techniques for neurodegenerative diseases during CNS demyelination.

## 5 NEUROMYELITIS OPTICA

NMO is an inflammatory CNS demyelinating disorder, which causes paralysis and vision loss induced by antibodies against the aquaporin‐4 expressed in astrocytes. In NMO, ON is the cause of visual impairment. Fundoscopy indicates vascular changes and disc atrophy with frosting (Bennett et al., 2015). During an acute attack, 80% of NMO eyes exhibit a severely decreased visual acuity (<20/200), and most suffering serious permanent vision loss based on incomplete recovery with higher axonal destruction (average 31.1 μm) than MS as calculated by OCT (Green & Cree, 2009). Intriguingly, the OCT sequence of RNFL thinning follow the ON is different as compared to MS, which provides a global pattern of loss with less predilection for the temporal quadrant (Bennett et al., 2015; Schneider et al., 2013). Small diameter axons are likely spared due to their dimensions (Petzold et al., 2010). Both the vascular changes of the fundoscopy (attenuation of the peripapillary vascular tree and the focal arteriolar narrowing) and the OCT pattern in the NMO show the potential of vascularly induced disruption in NMO (Green & Cree, 2009). Fellow eyes without the past background of ON do not show thinning of RNFL (Bennett et al., 2015; Ratchford et al., 2009). In NMO patients, recent studies have indicated that perifoveal modifications and morphological foveal, confirmed by high‐level expression of aquaporin‐4 channels in the Müller cells (Oertel et al., 2017; Shen et al., 2019).

## 6 EXPERIMENTAL AUTOIMMUNE ENCEPHALOMYELITIS

EAE is the most frequently applied experimental model that comprises all three main clinical manifestations of MS such as demyelination, inflammation, and axonal loss with a decreased demyelinating part.

EAE induction methods are highly heterogeneous and provide a significant effect on disease pathology as well as response to a variety of therapies. Traditionally, an adjuvant that includes bacterial components including attenuated *Mycobacterium tuberculosis* and stimulates an innate immune response sensitizes EAE animals to delivered myelin antigens such as MOG, oligodendrocyte‐specific protein, myelin basic protein, and proteolipid protein (Kaushansky et al., 2007; Quinn et al., 2011; Shindler et al., 2006, 2008; Smith et al., 2016). Such as, after immunization of proteolipid protein, the first attack of the relapsing‐remitting disease episode onsets as early as 9 days after postinduction (dpi) and peaks at about 12 to 15 dpi. From day 11, 41.5% exhibits bilateral ON and 43.9% unilateral ON, while 14.6% of animals do not indicate inflammatory infiltrate consistent with ON (Shindler et al., 2006, 2008). The MOG‐induced EAE may have a similar time‐onset, but in comparison, the disease is typically monophasic, also with disease peak (approximately ca. 15‐25 dpi) later, and the optic nerve inflammation usually bilateral (Quinn et al., 2011). Furthermore, the demyelination and neurodegeneration‐onset can also similarly occur as soon as 11 dpi. In EAE models, the severity of inflammation is associated with further demyelination as well as loss of RGC in the afferent visual system (Quinn et al., 2011; Shindler et al., 2006, 2008), the distribution and time frame of disease components, while several other factors, such as antigen concentration or mouse strain, also tend to affect it (Cruz‐Herranz et al., 2019; Soares et al., 2013). Particularly in transgenic mouse strains, the comparison of EAE induction could be restricted by the changed expression of the targeted protein or different composition of myelin (Guo et al., 2010). Spontaneous severely affected transgenic EAE models that carry myelin‐specific T‐cell receptors or by passive transfer of encephalitogenic T cells have further expanded the range of EAE models (Bettelli et al., 2003; de Rosbo et al., 2004; Guan et al., 2006; Pöllinger et al., 2009; Shao et al., 2004). However, the majority of these models are based on the MOG‐specific T‐cell reactivity (Bennett et al., 2015; Green & Cree, 2009; Ratchford et al., 2009; Schneider et al., 2013), the immunization with the myelin‐associated oligodendrocytic basic protein generates the CD4^+^ T cells also reportedly revealed chronic and severe EAE related with severe perivascular, demyelination, parenchymal inflammation, and neurodegeneration (Table 1).

**TABLE 1:** EAE models showing ON

Strains	Immunizations	Characteristics
TCR2D2	Pertussis toxin	TCR2D2 mice are more likely to develop spontaneous ON rather than to develop spontaneous EAE. Pertussis toxin injection increases the incidence of EAE and ON
C57BL/6J	Myelin oligodendrocyte glycoprotein MOG35‐55	Commonly monophasic, with a peak of disease ca. 15‐25 dpi and optic nerve inflammation are mostly bilateral
SJL/J	Myelin‐associated oligodendrocytic basic protein	Chronic EAE is associated with intense parenchymal and perivascular infiltrations, remarkable ON, axonal loss, and widespread demyelination
C57BL/6J	Passive transfer of the encephalitogenic T cells	The phenotype is highly inflammatory, while ON is most commonly bilateral
SJL/J	Oligodendrocyte‐specific protein	Chronic relapsing EAE with the intense parenchymal and perivascular inflammatory infiltrates, remarkable ON, axonal loss, and widespread demyelination
SJL/J	Proteolipid protein	The first attack of the relapsing‐remitting disease course starts ca. 9 dpi and peaks ca. 12‐15 dpi. From day 11, 43.9% of animals exhibit unilateral ON, 41.5% exhibit bilateral ON, and only 14.6% do not exhibit ON

EAE, experimental autoimmune encephalomyelitis; ON, optic neuritis.

### 6.1 Inflammation in experimental autoimmune encephalomyelitis

**FIGURE 1:**
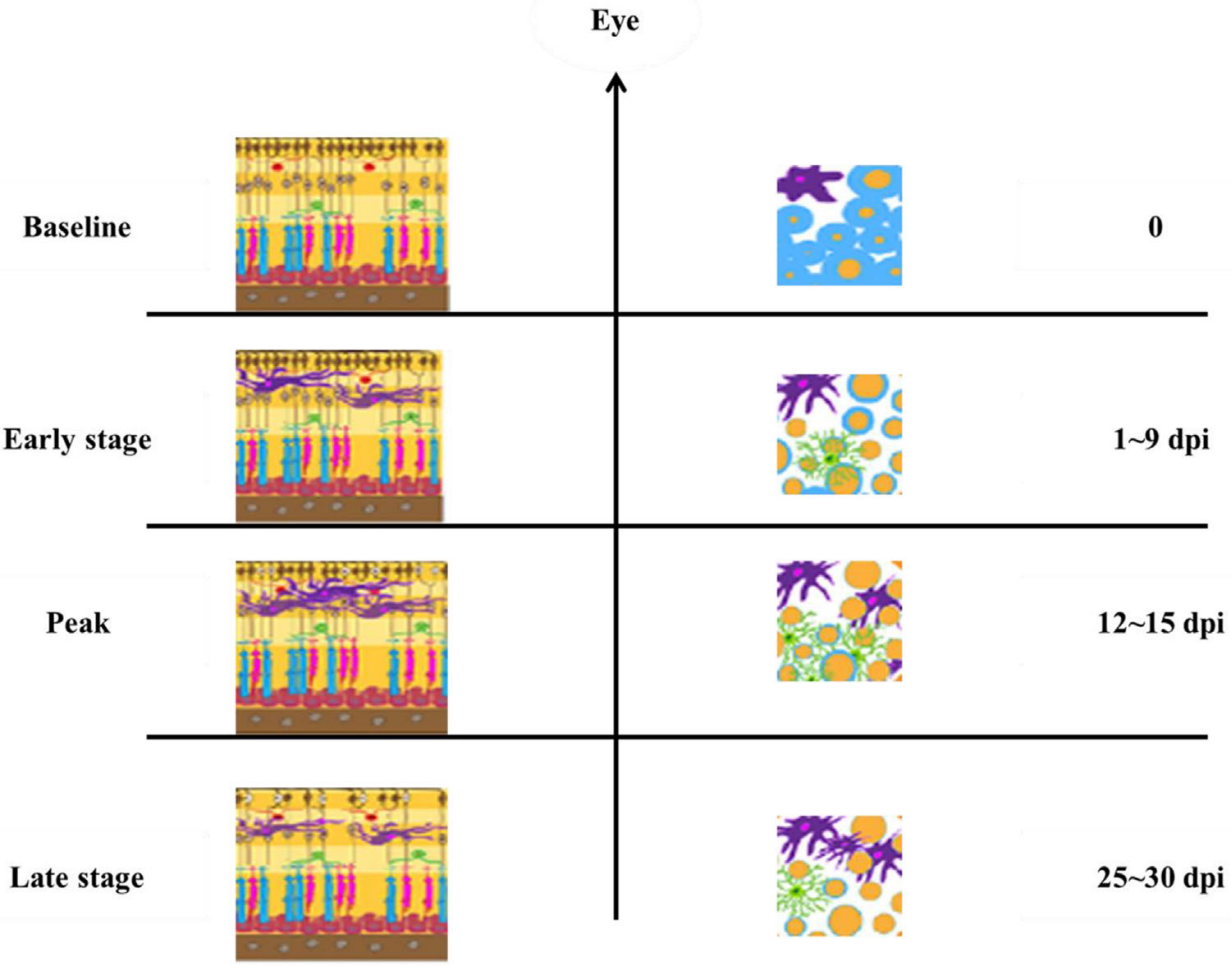
Retinal and optic nerve inflammation in the course of experimental autoimmune encephalomyelitis. Baseline characteristics include continuous layers of retinal ganglion cells, well‐structured myelinated axons in the optic nerve, resting microglia as well as astrocytes. The early and peak stage of disease includes activation of microglia and astrocyte, the polarization of macrophage, and T‐cell infiltration. Moreover, demyelination of axons, swelling, and early degeneration. Late stage include microgliosis, astrogliosis, T‐cell infiltration, axonal loss, as well as demyelination

In EAE, inflammations are generated by CD4^+^ T cells and microglia, while Th17 and Th1 cells play a key role (Horstmann et al., 2016). The continuous layered RGCs of the retina and myelinated axons of the optic nerve are arranged in a healthy mice, along with resting astrocytes and microglia (Figure 1). In the early stages of EAE, a blood–brain barrier is interrupted, microglial activation and the invasion of CD4^+^ T cells create an inflammatory CNS environment that causes the inner retinal layers to swell as well as optic nerve demyelination (Jin et al., 2019; Manogaran et al., 2019). The separation of peripheral immune cells from the glial cell response is still challenging, particularly because both reactions are temporal overlapping, and activated glia promotes the secondary recruiting of peripheral immune cells via chemokine releases. In subsequent EAE stages, the inflammatory combined reaction results in major demyelination, apoptosis, and depletion of up to half RGCs without a different retinal pattern (Shindler et al., 2006). From seven dpi, an increase in the number of active microglia is observable, particularly in the inner plexiform layer of the retina as well as ganglion cell, and continues to exist until the late stages of the disease (Horstmann et al., 2016, 2013; Jin et al., 2019; Manogaran et al., 2019, 2018). At 11‐15 dpi, the greater increasing trend in microglia appears to correlate with a major general cellular infiltrate as well as activation of astrocytes and macrophages through the release of proinflammatory markers such as IL‐6 (de Rosbo et al., 2004; Horstmann et al., 2016; Jin et al., 2019). In disease‐peak, microglia is also the main producer of inducible nitric oxide synthase, which leads to the inflammatory state (Jin et al., 2019). Massive infiltration of T cell begins mostly around the optic nerve head at 9 to 16 dpi and gradually reduces over the episode of the disease (Jin et al., 2019; Knier et al., 2015; Manogaran et al., 2019, 2018). It is reported that the inflammatory response in EAE could be area‐specific, with a higher frequency of centripetally infiltrating granulocytes, a lower frequency of macrophages, and IL17‐producing CD4^+^ Th cells in the optic nerve when compared to the spinal cord and brain (Knier et al., 2015). Aside from Th17 cells, IL‐17 appears to be generated by a significantly enhanced subset of γδ T cells in the optic nerve, unconventional T cells having characteristics of both adaptive and innate immune cells which are reported to play a crucial role in facilitating and sustaining EAE inflammation (Malik et al., 2016). In the EAEON, IL‐17 neutralization prevented thinning of the inner retinal layer and infiltration of the immune cell, which further highlights the effectiveness of the IL‐17 regulated mechanism (Knier et al., 2015; Larabee et al., 2016). In the early stage of inflammatory EAE, macrophages polarize into a proinflammatory phenotype which is the expression of increased levels of proinflammatory markers, such as IL‐23, IL‐12, CXCL‐11, and CXCL‐10, leading to an inflow of more cellular stress and inflammatory immune cells, while the proportion of anti‐inflammatory macrophages is reduced. It has been suggested in a recent study that fatty acids include omega‐6 and omega‐3, may promote a change of macrophages to an anti‐inflammatory form by increasing STAT3 phosphorylation and reducing NF/kB phosphorylation (Locri et al., 2018). The anti‐inflammatory phenotype is primarily determined by the expression of anti‐inflammatory cytokine IL‐10, which has independently shown the subsequent microglial infiltration, optic nerve demyelination, and MHC II expression (Matsuda et al., 2012). Several analyses have further explored the contributions of B cells, CD8+ T cells, and monocytes toward the pathology of EAE, but particular descriptions are still confirmed in the afferent visual system (Bullard et al., 2007; Hjelmström et al., 1998; Koh et al., 1992).

## 7 OPTICAL COHERENCE TOMOGRAPHY AS A BIOMARKER IN MULTIPLE SCLEROSIS

The OCT method allows the imagining of RGC injury and axonal loss. In 1991, Huang et al. described the first time that it is a novel technology for cross‐sectional tissue imaging from the Fujimoto Laboratory at the Massachusetts Institute of Technology in the USA (Frohman et al., 2005). Subsequently, the development was fast and spectacular. The first in vivo measurements of human cross‐sections of the retina were taken by the University of Vienna in 1993. The company Carl Zeiss Meditec launched the first commercially available TdOCT in 1996. Then the Institute of Physics at the Nicolaus Copernicus University, Poland, started enters the stage. In 1999, the first spectral‐domain optical coherence tomography (SOCT) for in vivo retinal imaging was established. In 2002, the same researchers obtained the first in vivo tomograms of the human eye (Theodossiadis & Grigoropoulos Vlassis, 2010; M. Wojtkowski et al., 2002; Maciej Wojtkowski et al., 2002). Basically, OCT is the same as an ultrasonography examination.

The distinction is that light waves (rather than sound waves) are used for tissue penetration. The wave return period is determined the same as in ultrasound after being expressed and distributed in the imaged tissue. Since the scanning beam velocity is extremely high (the light wave velocity is approximately 300,000 km/s, when compared to the velocity of sound in the water of 1500 m/s), direct assessment of the wave return period is practically impossible. Alternatively, wave interference is used for these calculations. According to the measuring process, OCT is divided into two types, such as the originally developed TdOCT and a newer SOCT. The reference mirror can be movable in TdOCT. It has been possible to compare the reflection of the light beam by the mirror to the light beam, which returns from the eye by changing its distance from the photodetector. It allows an accurate evaluation of the thickness and distances of individual tissue structures. SOCT does not have a moving reference mirror, while the diffractive grid is introduced and measures the light beam in different ways using the Fourier transformation (M. Wojtkowski et al., 2003). As a result, the SOCT images have higher resolution, and the acquisition time of a single 1024‐point line (a single A‐scan) is only 19 microseconds, which is 250‐fold shorter than TdOCT.

## 8 TDOCT VER. SOCT IN RETINAL EVALUATION

**FIGURE 2:**
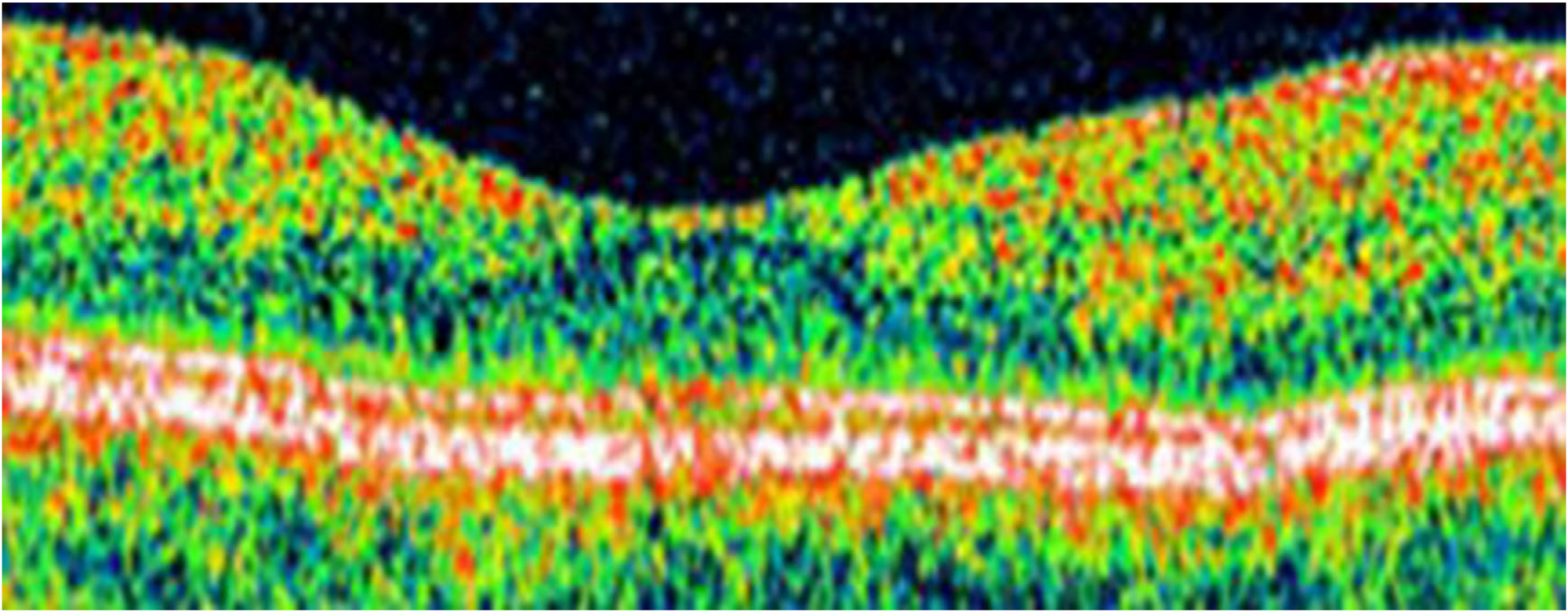
TdOCT Startus: the cross‐section of the macula

**FIGURE 3:**
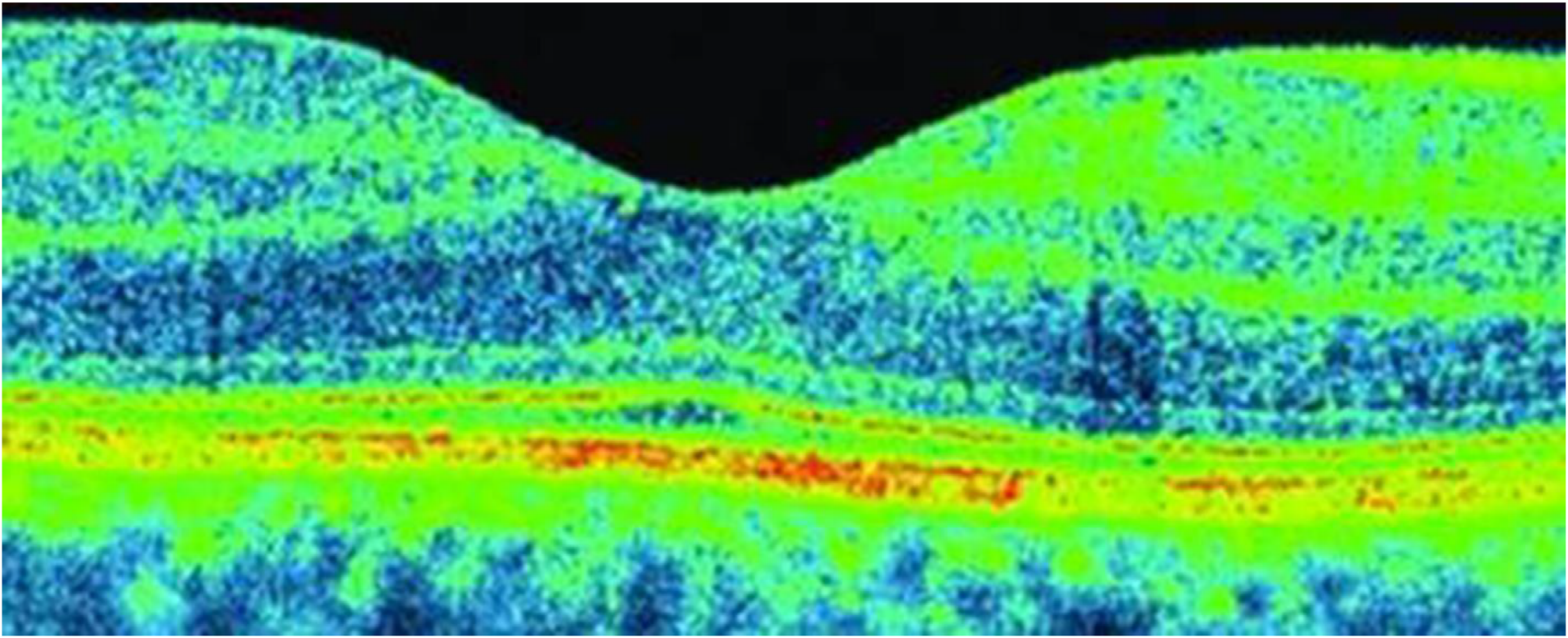
SOCT Copernicus HR: the cross‐section of the macula

TdOCT is a process that provides the acquisition of lower resolution retinal tomograms, as compared to the SOCT, with a considerably longer duration of the procedure. TdOCT tools used in ophthalmology have an axial resolution of approximately 10 mm and a transverse resolution of approximately 20 mm (Figure 2), while SOCT Copernicus HR (Optopol, Poland) has an axial resolution of 3 mm as well as a transverse resolution ranging about 12–18 mm (Figure 3). TdOCT is usually acquired up to 500 A‐scans per second, while Copernicus HR obtains 52,000 A‐scans per second (Nowińska et al.). Because of the high resolution of SOCT and also the short analysis time, which eliminates eye movement‐associated artifacts, while high‐quality scans can be obtained, enabling accurate morphological evaluation of the examined tissue as well as the acquisition of its 3D images. Furthermore, the procedure is noninvasive, contactless, and reproducible, which leads to its frequent use. OCT is also referred to as optical biopsy due to its high resolution and accuracy, as the retinal structure shown in the scans provides a high correlation to its real histology.

RNFL thickness was first measured using TdOCT on the ring with a diameter of 3.4 mm as a single peripapillary scanning. In 2002, triple circular scans of the same size were introduced due to low accuracy of measurement and low reproducibility of data. The final result of the examination was the mean of the three measurements. Since the start of the SOCT, the complete optical disc and the peripapillary region are scanned, while the images are accurately analyzed after the automated centration. In all situations involving nerve fiber loss, this imaging and the result processing technology greatly improved the reproducibility and reliability of the test, which leads to the frequent use of SOCT. SOCT is specifically effective in diagnosing and monitoring optic nerve diseases because of optical disc analysis and accurate measurement of RNFL. It is used in MS, glaucoma, and many other diseases which cause optic nerve damage such as post‐traumatic injuries, ischemia, brain tumors, Parkinson's, and Alzheimer's diseases. In terms of results acquisition resolution, speed, and reproducibility, the novel spectral domain imaging technique is superior to the traditional technique. In 55 MS patients, Bock et al. compared the two approaches. The result of the two tools is not exchangeable (Bock et al., 2010).

## 9 TDOCT AND SOCT IN RNFL EVALUATION IN MS

In 1999, in MS patients, the first study was published on evaluating the RNFL using TdOCT. Parisi et al. investigated 14 patients with MS, also with a history of previous retrobulbar ON, and 14 healthy individuals (Parisi et al., 1999). In MS patients, the RNFL was examined both in the eyes with the history of previous retrobulbar ON and eyes without previous episodes of ON. The mean RNFL thinning in eyes with the ON history was 46 and 28%, respectively, as compared with the control group and MS patients without ON history. In these patients, RNFL thickness was also decreased by 26% to the same assessments in the healthy control. It is discovered that RNFL thinning in the eyes with a history of ON could be due to axonal loss in the optic nerve (Trip et al., 2005, 2006). It has been reported that the pRNFL is thinning in eyes with a history of ON when compared to both MS patients and healthy individuals without a history of ON (Albrecht et al., 2007; Klistorner et al., 2008; Merle et al., 2008; Pulicken et al., 2007). According to different reports, RNFL thinning has also been confirmed in patients with MS who did not experience previous retrobulbar ON (Fisher et al., 2006; Pulicken et al., 2007). However, findings also suggest that statistically important RNFL decline appears in the natural course of MS both in eyes with and also without a history of previous ON. The SOCT study shows the mean pRNFL thickness (TSNIT), the RNFL thickness in individual sectors (expressed as numbers as well as on a graph with the specified normative values), and also the optic disc parameters. The observable defects including a reduced TSNIT graph as well as RNFL thinning in specific sectors of the right eye with ON history and also a reduced TSNIT in the left eye. The mean RNFL thinning in the case with a history of ON was 23.3% and without a history of ON was 9.5%, respectively (Kucharczuk, 2012).

Reich et al. reported during one of their first findings an association between optic radiation defect in MRI and RNFL atrophy in the respective eye (Reich et al., 2009). Furthermore, it is confirmed that it is obvious evidence of secondary, trans‐synaptic defect to ganglion cell axons and pRNFL thinning caused by the degenerative process, which includes the optic tract also in eyes without any clinical episodes of ON. In another analysis, Gordon‐Lipkin et al. (2007). studied 40 MS patients and identified a correlation between MRI‐confirmed cerebral atrophy and pRNFL atrophy. Moreover, the assessment of RNFL thickness with the use of OCT enables accurate evidence about the neurodegenerative brain lesions that occur in MS. These findings are associated with the results of other researchers, who reported that pRNFL atrophy develops both in eyes following previous ON as well as the eyes of MS patients who have no history of ON and that it corresponds with MRI‐confirmed brain atrophy (Albrecht et al., 2007; Burkholder et al., 2009; Fisher et al., 2006; Henderson et al., 2008; Lamirel et al., 2010; Ratchford et al., 2009; Siger et al., 2008; Trip et al., 2005).

Furthermore, in MS, results on the relationship of OCT measurements, grey as well as white matter volumes, are conflicting. It is reported that in early MS, OCT measurements of retinal atrophy are associated with volumetric changes in the white matter compartment but not the grey matter compartment as measured by MRI (Young et al., 2013). The correlation of RNFL thickness and TMV with brain parenchymal fraction is assessed in a broad prospective analysis of 104 RRMS patients (Dörr et al., 2011). Furthermore, RNFL thickness was related to the disease duration but not to the severity of the disease or patient age. On the other hand, brain parenchymal fraction was linked with severity rather than disease duration and was confounded by age. TMV was not linked to any of these factors. RNFL thickness could be a better parameter to monitor axonal injury longitudinally. In MS, it is assessed that the link between the harm to the anterior and posterior visual pathway is the evidence of trans‐synaptic degeneration (Gabilondo et al., 2014). The voxel‐based morphometry indicated that the RNFL thickness was directly related to the lesions in optic radiation of study inclusion and with visual cortex atrophy. In patients of severe prior ON, the mean visual cortex volume was lower than the patients without ON. The association of the gray and white brain volume with GCL and pRNFL was investigated in 63 RRMS patients (Zimmermann et al., 2013). In eyes without the previous ON, both parameters, such as GCL and RNFL, are comparatively associated with the entire brain and the gray as well as white matter atrophy. This relationship is interfered with by an ON event. Optic radiation thickness, optic radiation volume, OCT, 75 MRI, and visually expressed potentials were analyzed in 30 patients (26 RRMS and 4 CIS). In MS patients, both optic radiation integrity loss and anterior visual pathway damage observable by MRI are usual findings (Sinnecker et al., 2015). In 107 patients of MS, that dynamic changes, including OCT (automated macular segmentation) and MRI (brain structure volumetrics), were reported biannually (median follow‐up 46 months) (Saidha et al., 2015). In progressive MS, the GCIP and whole‐brain atrophy rates (white and gray matter) were closely associated rather than the RRMS. The use of OCT in research trials for clinical monitoring was supported. In MS, diffusion tensor imaging was used to determine the association between RNFL thickness and white matter damage. The changes in the RNFL suggest white matter damage that exceeds the visual pathway (Scheel et al., 2014). SOCT provides a reliable assessment of RNFL atrophy progression due to the accuracy and reproducibility of the pRNFL thickness determination. As a result, both the rate of neurodegeneration and the reaction to treatment can be assessed.

It is worth mentioning that RNFL thinning is not pathognomonic for optic nerve atrophy secondary to MS. It is reported that pRNFL thinning is associated with all forms of optic neuropathy, whether ischaemic, post‐traumatic, toxic, or inflammatory unrelated to MS (Choi et al., 2008).

## 10 SOCT FOR MACULAR EVALUATION

**FIGURE 4:**
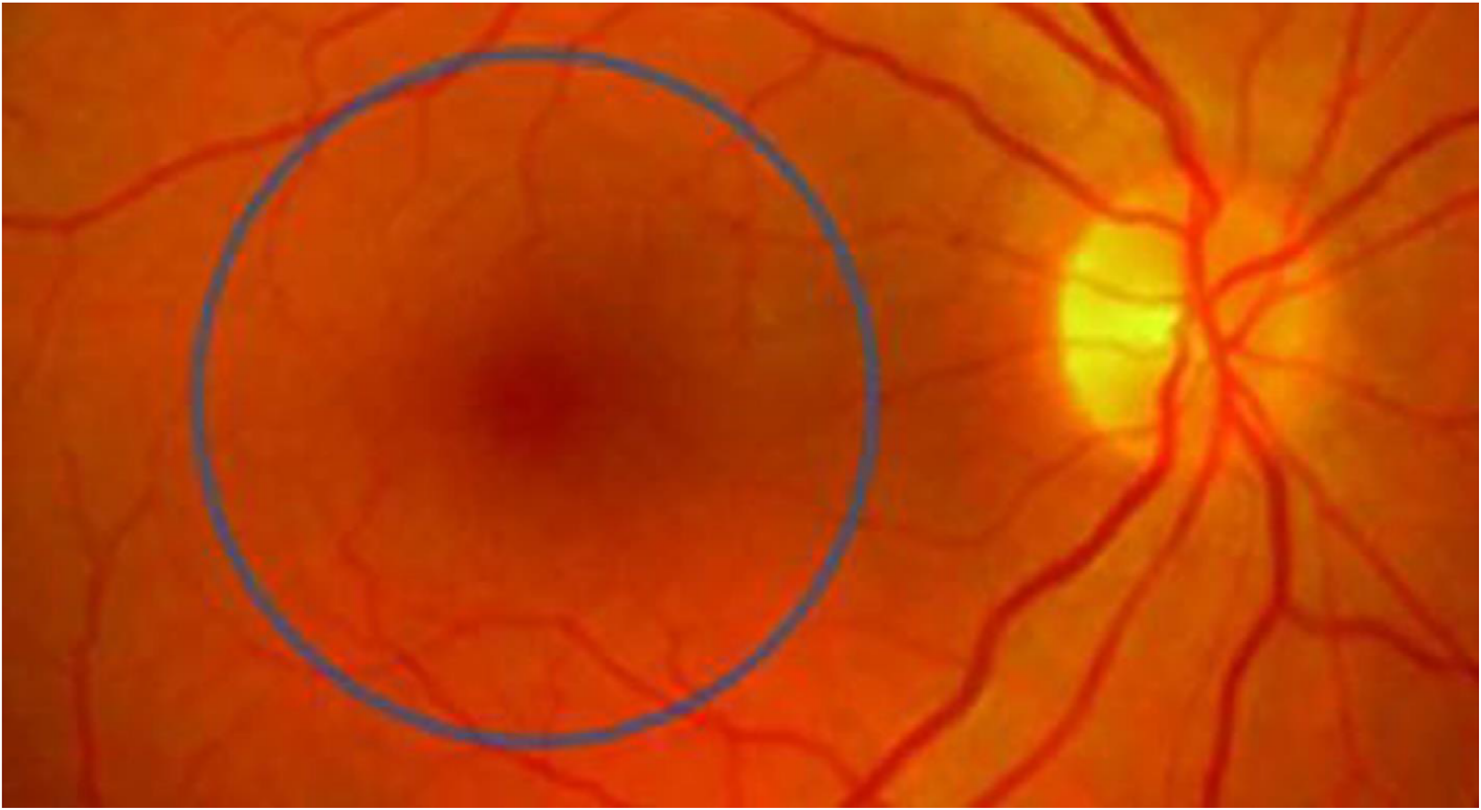
Fundus shows the optic disc and macula (circled area) with the fovea in the center

The retinal area is temporarily located to the optic disc known as the macula and represents further interest for neuro‐ophthalmologists investigating MS. It includes the central portion (approximately 2.85 mm) of the retina and has a natural pit in the center called the fovea (Figure 4). The perifoveal area, approximately 0.5 mm, is the only area of the retina in which the GCL consists of 5–7 cell layers instead of a single cell layer, as is the case in the rest of the retina (Burkholder et al., 2009). As a result, this area is an ideal target for studying neurodegeneration processes.

**FIGURE 5:**
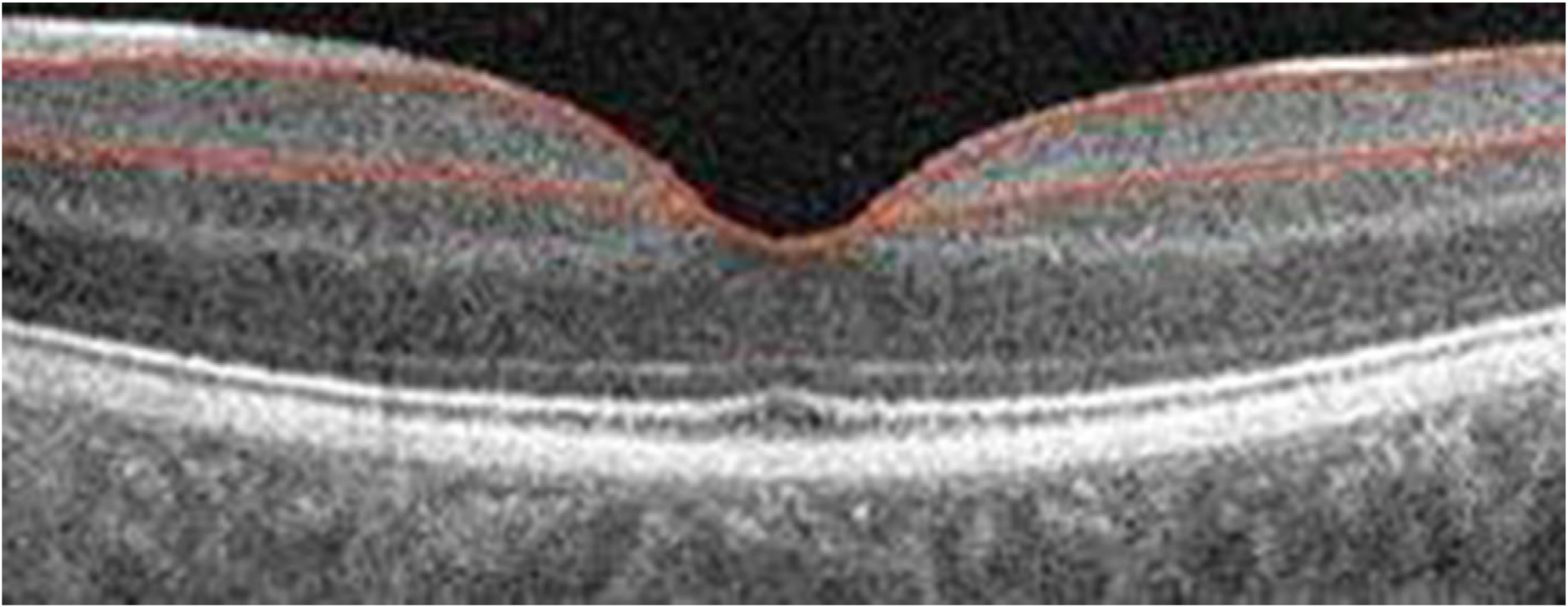
SOCT Copernicus HR: cross‐section of the macula shows the marked inner plexiform layer and ganglion cell layer, which form the ganglion cell complex

SOCT evaluates the TMV as one of the parameters in the perimacular region. The visible abnormalities include a significant reduction in TMV and retinal thinning in peripheral measurement rings of the right eye, along with the history of previous ON. The first TMV studies were reported in 2005. Trip et al. studied 25 patients along with a history of ON followed by an incomplete visual recovery. In eyes with ON history, the TMV was statistically lower when compared to eyes without the previous ON or controls (Trip et al., 2005). Findings also show that MS induces a reduction of TMV, which is significant in eyes with previous ON history. Furthermore, even in the eyes without previous ON, this reduction is present and still statistically important (Kucharczuk, 2012). It has been confirmed that TMV decrease takes place both in eyes after previous ON and in the eyes of MS patients but without an ON history (Fisher et al., 2006; Henderson et al., 2008; Ratchford et al., 2009; Reich et al., 2009; Waxman & Black, 2007). In MS, diverse pathological processes, such as re‐ and demyelination, but also the degrees of neuronal and axonal damage can describe the highly heterogeneous disease course. In previous studies, the thickness of RNFL and TMV are compared between different types of MS. Recent analyses reported that SPMS has more severe atrophy of GCIP and RNFL, as compared to PPMS (Oberwahrenbrock et al., 2012; Ratchford et al., 2013; Saidha, Syc, Durbin, et al., 2011). It is confirmed that a specific subset of MS patients with disproportionate thinning of inner and outer nuclear layers could occur as a primary process independent of optical nerve pathology (Saidha, Syc, Ibrahim, et al., 2011). Patients with more inner and outer pathology tend to have a tendency for an accelerated rate of impairment development. Brandt et al. (2011) evaluated data from a large cohort of 370 MS patients (262 RRMS, 61 SPMS, 36 PPMS, and 11 CIS) and 71 healthy controls at three large academic MS centers in Germany using the latest‐generation SOCT method (Specralis OCT, Heidelberg Engineering, Germany). In this cohort, the suggested macular thinning predominant phenotypes and their frequency were also described. Intriguingly, only 6 of 17 (35.3%) of PPMS patients experienced the macular thinning predominant criterion compared to Saidha et al., who did not identify the macular thinning predominant phenotype in their PPMS cohort. This finding does not support the conclusion of the particular macular thinning predominant phenotype of OCT in MS. Patients who show macular thinning predominant phenotype when evaluated with Cirrus OCT (Carl Zeiss Meditec, Germany) might be reassessed with Spectralis OCT to evaluate whether variations in instruments and scanning methodologies could be important. In MS, the thinning of inner retinal layers (INL, ganglion cell complex [GCC], and RNFL) are severely affected by heterogeneous disease courses (Balk et al., 2014). It is reported that the size effect association between disease period and RNFL thickness or TMV in MS‐NON (no ON) eyes only (Oberwahrenbrock et al., 2012). Considering the particular retinal histology in the perimacular region, it seems reasonable to conclude that the changes in retinal thickness and TMV represent not only retinal nerve fiber loss but also ganglion cell loss. In MS, reductions of TMV indicate that the primary damage includes RGCs and subsequently followed by nerve fiber damage (axons). Because of the advancement of SOCT, research has been conducted further into possible segmentation of retinal layers in the perifoveal region, as well as the potential of assessing the macular GCC, which is mainly composed of GCIP and ganglion cells (Figure 5). In MS patients, the GCC thinning is statistically important as compared to controls, and it is associated with their visual acuity as well as the subjectively evaluated quality of life (Walter et al., 2012). GCC atrophy, which is normal in MS patients and NMO, was also observed in patients without a previous history of ON (Syc et al., 2012). This seems to support the postulate that the degenerative process in these diseases induces both neuronal and axonal damage in the retina, without the previous history of ON, which can lead to degeneration of pre‐existing pathology. The absence of edema in the retrobulbar ON within the inner plexiform layers and ganglion cell were other important findings of this analysis. Edema commonly occurs within the RNFL during the acute stage of ON and highly restricts neurodegeneration evaluation based on pRNFL thickness assessment. On the other hand, the measurement of GCC thickness is not limited by this restriction. However, it is superior to the RNFL thickness assessment in measuring retinal neurodegeneration. Furthermore, retinal changes in the eyes of patients with NMO spectrum disorders (NMOSD) were compared to matched RRMS patients and healthy controls (Schneider et al., 2013). In NMO, the thickness of the RNFL was reduced more significantly as compared to MS following ON. RNFL thinning was obviously temporal preponderance in MS‐ON eyes, while in NMO‐ON eyes, RNFL was reduced more evenly. In patients with NMO‐ON, the outer nuclear layer and the inner retinal layer were thicker rather than NMO without the ON, whereas microcystic macular oedema (MMO) was primarily the cause of these differences. Moreover, OCT is still inadequate to help differentiate both conditions clinically in a single patient. Several studies have shown that ON in NMO usually leads to severe GCL and RNFL thinning and development of MMO more frequently than in MS (Bennett et al., 2015). RNFL thinning also occurs when ON is absent in MS, while subclinical damage appears rare in NMO. OCT could be effective in differentiating NMO from MS. A severe GCC thinning was shown in patients with active and early MS. It is suggested that the severity of neurodegeneration in the CNS is reflected by MS retinal lesions (Ratchford et al., 2013). Current analyses also indicate that RNFL thinning and some other retinal layers observed in SOCT correspond with changes in brain volume secondary to MS, as shown in brain MRI (Saidha et al., 2013). However, they may be the sensitive markers of progression for evaluating neurodegeneration in MS patients. In MS patients, the SOCT revealed not only the retinal layer atrophy but also many other defects. In MMO, it is observed that approximately 5% of MS patients are not related to MS‐caused uveitis (Gelfand et al., 2012). In patients with a history of ON, small pseudocystic lesions were found in the INL, which is mainly composed of horizontal, amacrine, bipolar, and Muller cells. In MS patients, the presence of MMO can show inflammatory and degenerative demyelination. They were linked to increased disease severity and reduced visual acuity. It is reported that the presence of MMO as well as enhanced thickness of the INL in SOCT associate with relapses, the activity of MS, the onset of increasing Gd+ focal lesions in MRI, and disease progression (Saidha et al., 2012). Recently, findings showed that the antibodies to MOG in certain NMO IgG‐seronegative patients with neuroimaging and clinical symptoms of NMO or NMOSD present a variant of the opticospinal MS or acute disseminated encephalomyelitis. This presents the question of whether patients with MOG‐IgG positive and AQP4‐seronegative phenotypes should be categorized as NMOSD (Zamvil & Slavin, 2015). MOG antibodies were found in 9 of 23 AQP4 antibody‐negative patients along with NMO/NMOSD as compared to 1 of 17 MS patients and 0 of 52 controls. MOG antibody‐positive patients showed significant optic disc swelling while they were more likely to develop a rapid response to steroid therapy and relapse after steroid discontinuation as compared to MOG antibody‐negative patients. MOG antibodies are closely related to bilateral or recurrent ON (Ramanathan et al., 2014). Moreover, anti‐MOG antibodies are often linked to recurrent ON or chronic relapsing inflammatory ON (Chalmoukou et al., 2015).

## 11 LIMITATIONS OF OPTICAL COHERENCE TOMOGRAPHY AS A BIOMARKER IN MS

However, in MS, like several other surrogate markers, OCT has some limitations that should be considered if the technology is to gain interest in the MS disease. In MS, as a surrogate endpoint, OCT lacks precision. Changes in GCIP, MV, RNFL, and INL structure determined by OCT must be examined in the context of the ophthalmic history of the patient. Numerous primary ophthalmic diseases may affect microcystic ME emergence in INL of MS patients, while these can be acknowledged when making conclusions about INL assessments and disease control. Such as, in a recent analysis, treatment of fingolimod was related to higher MVs related to eyes of MS patients who do not receive this drug (Nolan et al., 2013). Since fingolimod induces cystic ME in 1% of treated patients, it is likely that a milder type of drug‐induced ME led to the enhanced MVs found in MS patients taking the drug (Dinkin & Paul, 2013).

Additionally, higher MVs in the fingolimod group could not indicate any side‐effect of the drug while representing a lower efficiency of the drug in the prevention of MS‐related subclinical microcystic ME as compared to alternative treatments. Moreover, in this study, since more than half of the fingolimod patients received treatment due to MS‐related flares, it is possible that recent disease incidence, instead of fingolimod treatment, caused enhanced MVs in these patients relative to the control group. This is only one case scenario of how drug side‐effects might be mistaken as clinical value, as well as the possible drawbacks of OCT as a surrogate marker for neuroprotection in MS patients.

It is confirmed that OCT measurements of retinal atrophy in MS patients indicate loss of retinal axons and GCs, but the researchers also clarified how other pathological processes affecting the inner retina in MS patients might lead to inaccurate assessment of OCT measures (Green et al., 2010). Particularly, gliosis and perivascular inflammation affecting the inner retinal vessels in the RNFL might contribute to increased retinal layer thickness that does not indicate neuroaxonal swelling or expansion since OCT relies on presumed changes in tissue reflectivity at the interface of different retinal layers to segment retinal layers, while OCT measurements in MS patients can be evaluated with extreme care. Especially in MS patients, RNFL measurements cannot be assumed to show pure assessments of axonal integrity in the anterior visual pathway, as we do not know how the backscatter of low‐coherence infrared light used during OCT is changed by retinal inflammation or gliosis. However, with the advancement of OCT technology, it is possible to diagnose retinal pathology and apply software segmentation algorithms.

In addition to technology‐inherent problems, the assessing populations of MS patients are often challenged, which are heterogeneous by nature. Therefore, it is significant to make accurate interpretations by considering factors known to affect OCT measurements (including sex, age, high myopia, history of ON, coexisting ocular diseases, and use of medications). However, it would be difficult to escape the risks of clinical OCT dissociation, which has hindered the use of MRI as a biomarker in MS. Furthermore, critical analysis is also needed when making inferences about longitudinal changes in OCT measures, specifically when the magnitude of change being defined is in the test‐retest variability of the OCT being applied. It is reported that in MS patients, results from six longitudinal OCT analyses and found differences in the recorded rates of RNFL atrophy (Petzold et al., 2017). In 381 MS patients, the annual atrophy rate was −1.4 μm/year (Talman et al., 2010), which was similar to the results (−1.49 μm/year, n = 96) (Narayanan et al., 2014).

In comparison, numerous subsequent analyses revealed the annual rates of RNFL atrophy to be approximately third of the stated estimates, ranging from −0.36 μm/year (n = 107) to −0.53 μm/year (n = 168) (Petzold et al., 2017). In a study of 58 MS patients, no major RNFL changes were recorded over a period of 2 years (Serbecic et al., 2011). The differences in these reports could be described by the demographic differences in the study of populations.

MS patients without previous history of ON and a shorter period of disease showed the highest annual atrophy rate. Obviously, these findings could suggest an important phenomenon in subclinical neuroaxonal loss occurring early in MS. Additionally, the changes in GCIP and RNFL measures can be more observable for MS patients with shorter duration of disease, which have more intact neuroaxonal substrate relative to MS patients with longer duration of disease and pre‐existing injury to afferent visual pathways. In patients with subclinical CNS lesions, it can be difficult to identify severe GCIP and RNFL thinning in more advanced disease and with the OCT floor effect, limiting the identification of new neuroaxonal damage in the context of pre‐existing neuroaxonal damage. It is reported that MS patients with a longer duration of disease (above 20 years), problems with the calculation of incremental neuroaxonal loss in the presence of neuroaxonal damage can describe the so‐called plateau effect in the RNFL thickness (Balk et al., 2016).

Moreover, OCT presents a retinal structure measure but does not specifically consider cortex‐adaptive responses, which could affect the recovery potential of MS patients. Subsequently, clinical OCT dissociation can also take place, which is similar to the MRI measures, because of the different abilities of patients with MS to compensate for their age and stage of disease after injury to afferent visual pathways.

## 12 FUTURE TRENDS OF OPTICAL COHERENCE TOMOGRAPHY AS A BIOMARKER IN MS

The emerging evidence supports the use of OCT as a low‐cost, easily accessible biomarker in MS. Obviously, pathobiological mechanisms that connect OCT measurements of the retinal constituent to global CNS inflammatory effects, neurodegeneration, and axonal loss are possible in MS. OCT measurements are also safe and very stable on several possible machines. This innovation has provided current, large‐scale, and multicenter collaboration in research sites. However, when the OCT measurements are combined with standard visual outcome measurements, the accuracy of the technology in a well‐characterized population of patients is relatively reliable to monitor the temporal effects of the CNS inflammatory lesions. Moreover, the coherence of the technique is indicated by the associations of OCT and other frequently used measurements of MS disease (MRI‐determined T2, clinical relapses, gadolinium lesions, and NEDA‐3 parameters). In this context, OCT usually meets some specified criteria of the biomarker for diagnosing MS, monitoring the progression of the disease, and determining response to therapy.

## 13 CONCLUSIONS

SOCT analyses indicate that each case of MS involving pRNFL thinning followed by reduced TMV, regardless of the previous retrobulbar ON. The severity of these defects increases as the disease progresses, which represents the progressive degeneration of nerve fibers and RGCs. The changes in these parameters can be a noninvasive and sensitive indicator that is useful in the assessment of degeneration as well as inflammation in MS, as an alternative marker to monitor the treatment, which is currently being used in patients with relapsing‐remitting MS treated with disease‐modifying drugs.

## AUTHOR CONTRIBUTIONS

All the authors have accepted responsibility for the entire content of this submitted manuscript and approved submission.

## CONFLICT OF INTEREST

The authors declare that they have no conflict of interest.

### PEER REVIEW

The peer review history for this article is available at https://publons.com/publon/10.1002/brb3.2302.
